# Exploring the Impact of the Prescription Automatic Screening System in Health Care Services: Quasi-Experiment

**DOI:** 10.2196/11663

**Published:** 2019-06-14

**Authors:** Yan Li, Xitong Guo, Carol Hsu, Xiaoxiao Liu, Doug Vogel

**Affiliations:** 1 eHealth Research Institute School of Management Harbin Institute of Technology Harbin China; 2 Management Science and Engineering Tongji University Shanghai China

**Keywords:** prescription drug monitoring programs, hospital information system, quality of health care, medical errors

## Abstract

**Background:**

Hospitals have deployed various types of technologies to alleviate the problem of high medical costs. The cost of pharmaceuticals is one of the main drivers of medical costs. The Prescription Automatic Screening System (PASS) aims to monitor physicians’ prescribing behavior, which has the potential to decrease prescription errors and medical treatment costs. However, a substantial number of cases with unsatisfactory results related to the effects of PASS have been noted.

**Objective:**

The objectives of this study were to systematically explore the imperative role of PASS on hospitals’ prescription errors and medical treatment costs and examine its contingency factors to clarify the various factors associated with the effective use of PASS.

**Methods:**

To systematically examine the various effects of PASS, we adopted a quasi-experiment methodology by using a 2-year observation dataset from 2 hospitals in China. We then analyzed the data related to physicians’ prescriptions both before and after the deployment of PASS and eliminated influences from a variety of perplexing factors by utilizing a control hospital that did not use a PASS system. In total, 754 physicians were included in this experiment comprising 11,054 patients: 400 physicians in the treatment group and 354 physicians in the control group. This study was also preceded by a series of interviews, which were employed to identify moderators. Thereafter, we adopted propensity score matching integrated with difference-in-differences to isolate the effects of PASS.

**Results:**

The effects of PASS on prescription errors and medical treatment costs were all significant (error: 95% CI –0.40 to –0.11, *P*=.001; costs: 95% CI –0.75 to –0.12, *P*=.007). Pressure from organizational rules and workload decreased the effect of PASS on prescription errors (95% CI 0.18-0.39; *P*<.001) and medical treatment costs (95% CI 0.07-0.55; *P*=.01), respectively. We also suspected that other pressures (eg, clinical title and risk categories of illness) also impaired physicians’ attention to alerts from PASS. However, the effects of PASS did not change among physicians with a higher clinical title or when treating diseases demonstrating high risk. This may be attributed to the fact that these physicians will focus more on their patients in these situations, regardless of having access to an intelligent system.

**Conclusions:**

Although implementation of PASS decreases prescription errors and medical treatment costs, workload and organizational rules remain problematic, as they tend to impair the positive effects of auxiliary diagnosis systems on performance. This again highlights the importance of considering both technical and organizational issues to obtain the highest level of effectiveness when deploying information technology in hospitals.

## Introduction

### Background

Hospital information systems (HISs) have been adopted as effective tools to mitigate medical treatment costs and improve quality of care [1]. According to the report from Healthcare Information and Management Systems Society analytics, the rates of adoption of hospital intelligence solutions for the US health care market in 2017 increased up to 62% [2] and those for Chinese health care market during 2017-2018 increased up to 55% [3]. However, from the HIS users’ perspective, 36.36% of users show their dissatisfaction toward the effects of hospital information technology (HIT). This indicates that despite extensive investments on HISs, the effects of related systems remain somewhat questionable and controversial, in particular, under various environments and for various individuals [4].

Most HISs in China provide informative guidance to the decision makers, which further aim to support their judgments and adoption of the systems [5]. As a significant part of the HIS, the Prescription Automatic Screening System (PASS) focuses on rational drug usage (eg, drug interactions, pharmacologic antagonism, and chemical incompatibility) and searches for available drug information. Past literature posited that PASS could provide high-quality alerts for users [6] and is an effective approach to improve prescribing behavior [7]. Compared with other HISs (eg, electronic medical record [EMR] and HIS), PASS could directly help in the avoidance of adverse drug reactions (ADRs) and adverse drug events (ADEs) and in the reduction of high medical treatment costs caused by inappropriate drug usage [1,8]. Considering the benefits of PASS, many hospitals have already adopted this system. However, the effects of PASS and ways to improve the effectiveness of using PASS are still unclear [9]. Organizations generally develop related rules to enhance the positive effects of PASS, such as a weekly report of dosage; how these rules influence the effects of PASS on medical performance is less explored. Studies have also presented an insignificant correlation between the system in hospitals and prescription behavior [4,9]. Hence, exploring the factors that may influence the effectiveness of PASS implementation, such as individual characteristics and environmental factors, would contribute to further understanding the mechanism of effects of PASS.

### Research Context

**Figure 1:**
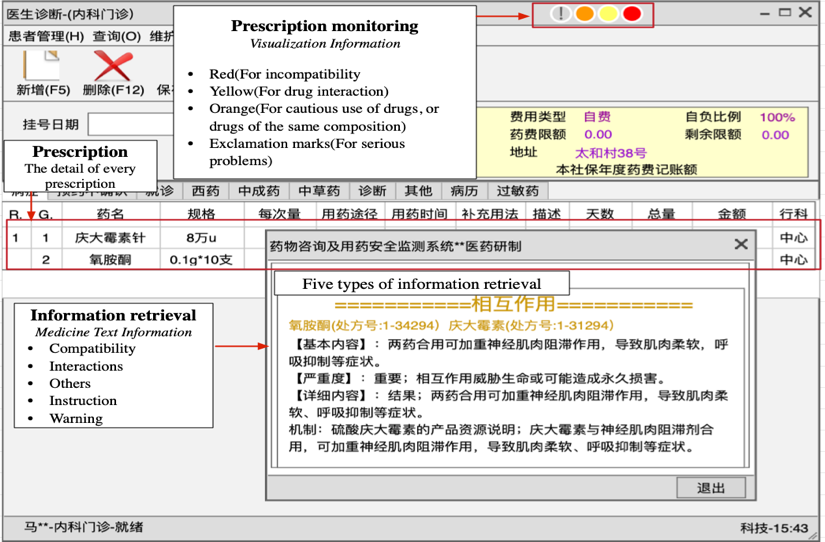
Example of the Prescription Automatic Screening System interface.

PASS is an aided diagnosis system that aims to monitor physicians’ prescription behavior via reminders to avoid the potential risks and uncertainties inherent in preparing the prescriptions. The primary affordances include *supporting the information retrieval* and *monitoring a prescription. Information retrieval* provides a related knowledge database for physicians to search for information when they are unsure about a situation. PASS provides the detailed usage information of medicines including incompatibility, interactions, drug instructions, warnings, and others. *Prescription monitoring* provides support for physicians to avoid repeated diagnoses, drug interactions, and medication contraindications and to ensure dosage control through the reminders. During the decision process, a knowledge-centered design system promotes more direct interactions between physicians and the system using differently colored alerting lights. These lights represent different risks and facilitate increased communication between physicians and patients, thus helping acquire more patient information to avoid ADRs/ADEs. Specifically, the level of risks existing in prescriptions is presented by these alerting lights, including exclamation marks (for serious problems), red (for incompatibility), yellow (for drug interactions), and orange (for cautious use of drugs, or drugs of the same composition). Such reminders are a form of *in-process* control and serve as the foundation for our subsequent statistical analysis. Figure 1 presents all the above-mentioned information in detail.

### Literature Review in Hospital Information Systems

Previous literature on HISs has 2 streams, with one focusing on the exploring factors related to HIS adoption [10] and the other focusing on the effects of implementing an HIS in medical institutions. Increasing number of empirical studies have explored the effects of HISs, with studies suggesting that HISs have inconsistent effects on medical performance, such as the medial cost and prescription errors [11,12]. To unfold the black box of the impact of HIS investment, studies further explored various types of HISs to investigate how HISs could improve the medical performance [12-14]. Studies also investigated the mediators between the HIS and performance to explore the underlying mechanism of HIS effects [12]. However, the HIS (eg, EMR) mainly improves the efficiency of physicians’ work, which then influences physicians’ performance. Limited studies explore the effects of systems such as PASS on physicians’ performance by using actual behavior data. Such systems (eg, PASS) have specific functions, such as monitoring and controlling, which differ those of the EMR. Besides, previous studies mainly adopted a descriptive analysis or regression model to explore the relationships in the literature on HIS effects; limited studies deployed a quasi-experiment to explore the effects of an HIS, which is a well-designed methodology to explore the causal relationships [12-14]. Hence, considering these research gaps, this study further explored the contingency factors related to PASS effects by a quasi-experiment in hospitals, which could be generalized to the literature of HIS effects.

### Theoretical Foundation

This study was theoretically based on the knowledge-based view [15] and pressure perspective [16]. As tacit technical knowledge of physicians is quite difficult to transfer to another individual [17], coordination among individuals who have expertise in various domains of knowledge becomes a key factor to enhance the quality of performance of hospitals [18]. To improve the coordination efficiency among different groups of individuals within the hospital setting, information technology (IT) provides a technical framework to facilitate the integration of knowledge among various individuals [19]. In this way, IT could not only promote effective knowledge exchange [20] but also provide a pathway for the mapping of knowledge to fulfill the knowledge gaps of a particular individual or group of individuals. Hence, benefiting from the knowledge integration, PASS could provide more information for physicians’ decision making, which could improve medical performance.

However, the effectiveness of the effects of knowledge-based view on decision making may vary when individuals face both internal and external pressures. Hence, to fulfill the research gaps of exploring contingency factors between PASS and medical performance, this study built a theoretical model from the pressure perspective and adopted the measurements based on theoretical literature and qualitative results from interviews with caregivers. Regarding the definition of pressure, pressure refers to “those organizational events which cause the individual anxiety, restlessness and irritation” [21]. Hence, in line with this theoretical logic, individual pressure refers to the psychological emotion coming from individual factors (eg, clinical title and perceived illness risks), whereas organizational pressure comes from organizational factors (eg, medical policy and workload). Given the psychological aspects, physicians generally make decisions based on the balance and appropriateness between evidence (eg, patients’ real-time conditions) and their cognition (eg, a domain of knowledge and previous experience) [22]. Furthermore, anchoring bias is quite common in the decision-making process, most particularly in situations with a high level of uncertainty regarding individual heterogeneity in both patients (eg, health evidence) and physicians (eg, cognition). Hence, under pressure from an individual and organization, the effects of PASS vary with various types of pressure by having different reactions to the implementation of PASS.

In the context of this study, PASS provides not only the basic functions for integrating knowledge from diverse sources (eg, information retrieval) but also supports the decision through the integration of knowledge presented through advanced technology (eg, alerting light). Despite the similarities of intelligent decision-making support between PASS and smart diagnoses, the visualization information derived from PASS could provide more efficiency in updating the knowledge of physicians to enhance their decision-making processes. For example, physicians will know what types of new drugs are inappropriate for specific cases by merely observing the alerting lights. Hence, based on the limited research exploring related topics, this study will investigate the correlation between the adoption of PASS and medical performance from the knowledge-based view.

To further explore the moderators in this context, we conducted several interviews with caregivers working in hospitals (ie, 3 physicians, 2 nurses, 4 IT employees, and 3 administrators), so as to further explore the actual usage behavior of PASS (details of interview results are provided in Multimedia Appendix 1). The findings of these interviews are presented as follows:

- Physicians may ignore the alerting information when working under high workload.
- The weekly report of medicine usage may influence physicians’ performance. In some hospitals, departments will have a statistic report of medicine usage for each physician every week. This report will calculate the specific dose of each medicine per physician, such as antibiotics.
- Physicians who have an elevated position and level of responsibility may have different opinions that may contradict the alerting information.
- Physicians may spend more time to make a decision when confronting cases with a high risk (details shown in Multimedia Appendix 1).

**Figure 2:**
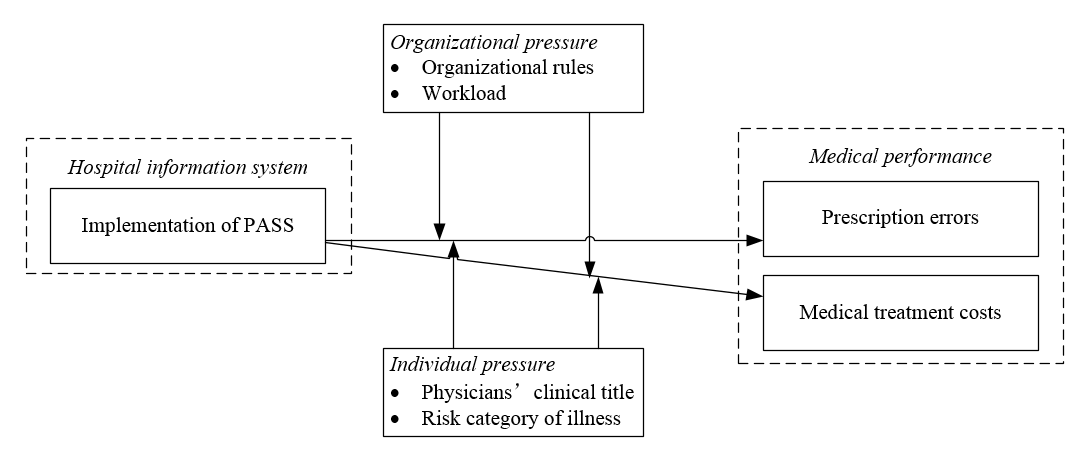
Framework of the Prescription Automatic Screening System (PASS) working principle.

On the basis of the above-mentioned findings, we identify 4 moderators: workload, organizational rules, clinical title, and risk of diseases. In line with the knowledge-based view and pressure theory, workload and organizational rules are organizational events that will create pressure on physicians; physicians’ clinical title and the risk of diseases are individual issues. High pressure may distract physicians’ attention, which, to some extent, will influence the effects of PASS on medical performance. For example, with high pressure of workload, physicians may accelerate the decision process and follow their anchoring cognitions, which may impair the effects of PASS. The research framework is shown in Figure 2.

The objectives of this study were to (1) explore the effects of PASS (an auxiliary system of the HIS) on prescription errors and medical treatment costs and (2) examine how the effects of PASS vary when physicians are under pressure from the organization (eg, a high workload and organizational rules from administrators) and individual (eg, clinical title and risk of diseases).

## Methods

### Experiment Design

**Figure 3:**
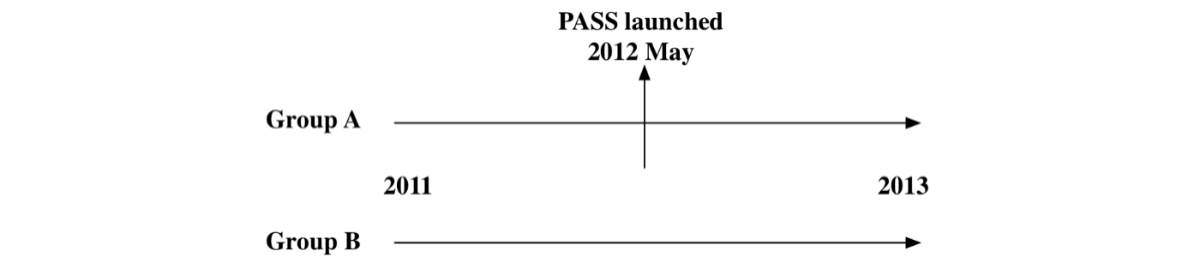
Research design. PASS: Prescription Automatic Screening System.

To test the proposed relationships, we used the quasi-experiment methodology in this study. We used 2 groups to compare the outcomes, that is, the control group and the treatment group. To be specific, in our context, we chose a hospital that had already deployed a PASS into practice as the treatment group (group A) and a hospital that had not deployed a PASS as the control group (group B) as presented in Figure 3.

There may exist endogeneity problems because of the effects of physician-specific unobserved factors on prescription errors and costs. To be specific, physicians’ individual preferences toward the system may simultaneously influence their using behavior of the system. Thus, to solve the endogeneity problems arising from the self-selection, this study adopted the propensity score matching (PSM) integrated with the difference-in-differences (DID) analysis as recommended by the current literature [23,24].

Furthermore, the methodology of DID estimates the difference in pre- and postbehavior or outcome differences between the 2 groups of physicians, the treatment group (ie, physicians who use PASS) and the control group (ie, physicians who do not use PASS). Because a comparison of the differences between pre- and postbehavior could not eliminate the extraneous factors, the DID provides a method to adopt the benchmark physicians who do not use the PASS to control the influence of extraneous factors. Thereafter, to eliminate the differences between groups, the physicians in these 2 groups should have similar individual features, such as similar clinical title and gender. Hence, this study used the PSM to match the similar physicians in 2 groups, which could impair the temporally invariant estimation bias and also simulate a randomized experimental setup [25].

Finally, the endogeneity problem may arise from the actual use behavior of physicians. First, because PASS is an assistant tool in HIS, caregivers will use it from the day the system is launched, as the implementation of this system is mandatory for hospitals. Furthermore, in the evidence of the interviews with physicians, IT employees, and administrators, their answers support that physicians adopt PASS while they are making prescriptions. Hence, the physicians in the treatment group actually use PASS.

### Data Collection

**Figure 4:**
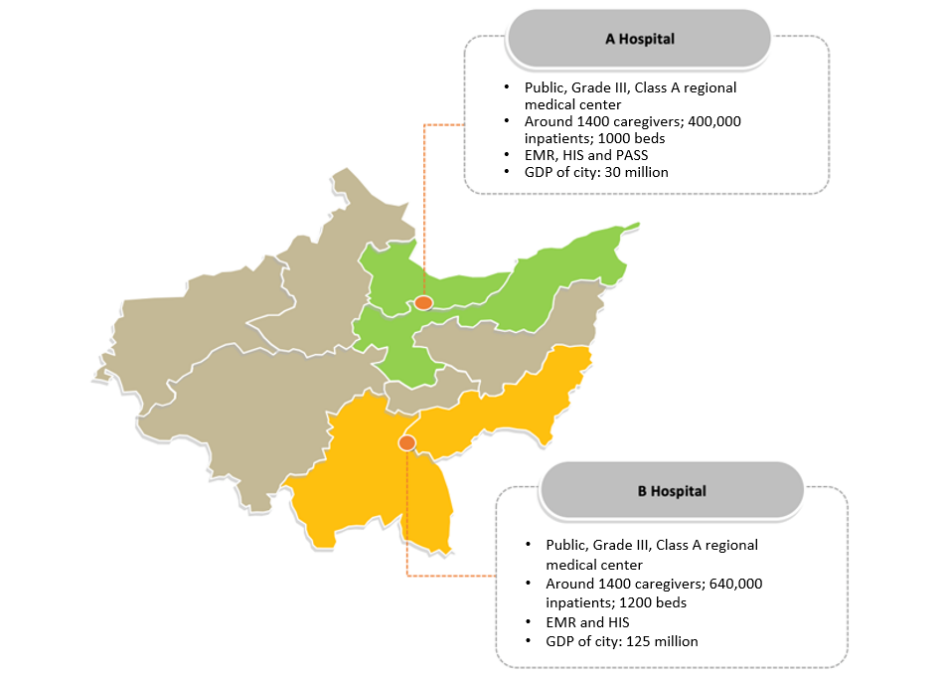
Hospital descriptions. EMR: electronic medical record; HIS: hospital information systems; PASS: Prescription Automatic Screening System; GDP: gross domestic product.

To test the model, we collected data from 2 hospitals in the same province in China. They are all public grade III hospitals, which are also the best hospitals in each city. They have similar features in basic medical conditions, technology facilities, and organizational environment. Specifically, both hospitals, have approximately 1000 medical caregivers and 0.5 million inpatients, and they make use of advanced HITs (ie, EMR and HIS). Moreover, these 2 hospitals are located in the same province having a similar natural environment, such as the cold weather, which causes respiratory illnesses in residents. The control hospital has a higher gross domestic product (GDP) than the treatment hospital, but this may have little impact on the experiment. This is because both hospitals invested similar advanced information systems; physicians and patients share similar economic status, which explains that GDP has little impact on physicians’ decisions and medical costs. Visualization of basic information has been shown in Figure 4. Robustness check also proves that the confounding effect is not from the differences between the 2 hospitals.

PASS deployed in hospital A is developed by 1 of the 2 biggest software firms. PASS provides information and decision support to physicians and pharmacists. To ensure the information quality, a database of PASS collects information (eg, medicine information, medical policy, and rules) from an authoritative medical dataset and updates every 2 years. Hence, PASS could provide reliable information for physicians to assist their decisions.

We chose the general inpatients as the sample frame of this study. This is because inpatients would be taking an array of medicines; as such, prescribing another medication could easily lead to incompatibility and interactions among them. Next, we used medicine usage information and medical information based on our selected hospitals in China during the period between 2011 and 2013. In group A, the hospital using a PASS, this system was introduced in May 2012, which enabled examination before and after system implementation of PASS.

According to descriptive statistics, the overall data included 754 physicians (400 in group A and 354 in group B), and 11,054 patients (5199 in group A and 5855 in group B). Group A included 55.5% (222/400) of male physicians and 44.5% (178/400) of female physicians, whereas group B included 58.2% (206/354) of male physicians and 41.8% (148/354) of female physicians. Moreover, our data indicated 26.7% (201/754) of high clinical title physicians (ie, chief physicians), 28.9% (218/754) of physicians of medium clinical title (ie, attending physicians), and 44.4% (335/754) of low clinical title physicians (ie, physicians).

### Variables Description

To measure medical performance, we adopted medical treatment costs and prescription errors as the dependent variables of this study. We calculated the medical treatment costs by ∑(a_ij_/n_i_), where a_ij_ denotes the fee of each medical category *j* for patient *i*. We then calculated the number of prescriptions withdrawn within 10 min, and we excluded the data of prescriptions that were withdrawn because of patients’ reasons. We assumed that withdrawing prescriptions within 10 min was unnecessary, which could be well avoided during the process of prescription making.

Hospitals usually implement relevant policy to ensure the effectiveness of information systems. Hence, we used a dummy variable to measure the organizational rules, that is, if the department implements rules related to the use of PASS, then the dummy variable is 1, otherwise the dummy variable is 0. We calculated the total number of patients per physician to measure the workload. Finally, based on a reference to the existing classification about the severity of illness, this study identified 4 categories (ie, 1-4) of illness risk by manual labeling [26]. Because the decisions of physicians depend partially on the individual’s characteristics, we used gender as the control variable as presented in Table 1.

**Table 1:** Variable definitions.

Variables		Symbols	Measurements
**Dependent variable**			
	Prescription errors	Error	The average number of prescriptions withdrawn within 10 min per physician in a month
	Medical treatment costs	Cost	The average medical costs per physician, ∑(a_ij_/n_i_)
**Independent variable: implementation of PASS^a^**			
	Time of PASS	InSys	The time of the system launched, 0=No, 1=Yes
	Treatment	Treatment	Whether hospitals implement the PASS, 0=No, 1=Yes
**Moderator: organizational pressure**			
	Workload	WorkLoad	The daily number of patients seen by a physician per month
	Organizational rules	Ins_pres	Whether the physicians stay in the department having a statistical report related to usage of PASS every month, 0=No, 1=Yes
**Moderator: individual pressure**			
	Risk category of illness	Risk	The risk category based on the case information, for example, readmission times, age, inpatient health condition, and ICD-10^b^
	Physicians’ clinical title	Title	Dummy variables of physicians’ clinical title, such as chief physician and attending physician
**Control variables**			
	Gender	Gender	Gender of physicians, that is, male or female

^a^PASS: Prescription Automatic Screening System.

^b^ICD-10: International Classification of Diseases, Tenth Revision.

### Model Specifications

#### Propensity Score Matching

To measure the causality between the system and user performance and to eliminate the sample differences between the 2 hospitals, we formed group A (the treatment group) and group B (the control group) using the PSM method to compare the effects. We created a statistical equivalence to balance all relevant characteristics that existed before the system launch [27]. Data before the intervention were available in both groups, and we used 12 months of data pertaining to patient characteristics for both groups before the launch of the PASS.

**Figure 5:**
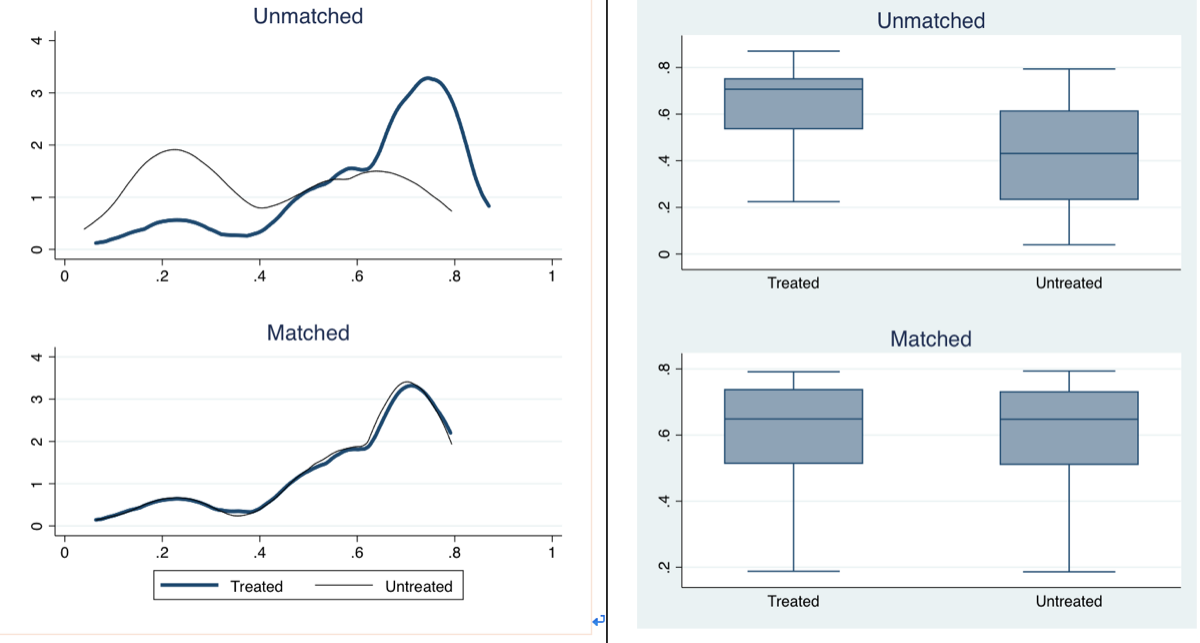
A visual analysis of propensity score distributions through box plots and histograms.

We used the kernel-based method in PSM. As physicians’ gender partly influences prescriptions, this reflects differences in attitude toward technology adoption [28]. In addition, the physicians’ clinical title and the number of patients per physician also play a role in physicians’ use intentions, as an overworked physician will possess lower work efficacy and face greater pressure. On the basis of the preceding section, we considered these variables as covariant variables (see Multimedia Appendix 1). After matching the 2 groups, we had 695 physicians. To check the substantial overlap in the characteristics of the physicians who adopted PASS and those who did not (ie, common support conditions), we conducted a visual analysis of the propensity score distributions through box plots and histograms (see Figure 5) and found evidence for the existence of common support.

#### Effects of Prescription Automatic Screening System on Prescription Errors and Medical Treatment Costs

This study combines the PSM and DID methods to verify the before and after effects of (1) prescription errors and (2) medical treatment costs. For treatment and control groups, the logarithm of the error of the prescription is modeled as follows:

Ln(Erro_it_) = δ_oj_ + δ_1_Treat_i_ + δ_2_InSys_it_ + δ_3_Treat_i_ × InSys_it_ + ΘX_i_ + ξ_it_ (1)

The independent variables in the equation of the other 2 dependent variables are the same. In equation 1, *i* denotes a treatment group or a control group physician and *t* denotes the time period. *Treat*_i_ is the treatment dummy variable (1 denotes that the physician is in the treatment group and 0 denotes that the physician is in the control group), whereas *InSys*_it_ is a dummy variable denoting the launch of PASS, taking the values 0 and 1 for periods before and after the system launch, respectively. For physicians belonging to the matched pair *i*, *X*_i_ represents a vector of control variables, with Θ being their corresponding estimated coefficients.

*δoj* refers to the physician-specific fixed effects that capture the differences in baseline relationship intensity, which enable the controlling of unobserved heterogeneity among physicians. It is to be noted that in the above formula consisting of matched treatments and control group physicians, monthly data that span both pre- and postlaunch time periods of PASS of this study are used (December 2011-December 2012), resulting in time-series data that are then stacked for estimation. The main parameter is *δ*_3_, which captures the changes in the average length of stay for treatment and physicians post adoption compared with physicians of the control group who did not adopt the system.

#### Moderating Effects of Individual and Organizational Pressure

Next, we described an alternative version of the model to the one presented previously (in equation 1), which enabled us to test the hypotheses posited earlier. Therefore, to investigate the impact of moderating variables, we used the following formula:

Ln(Error_it_) = γ_oj_ + γ_1_TreatD_i_ + γ_2_InSys_it_ + γ_3_TreatD_i_ × InSys_it_ + γ_4_ TreatD_i_ × Moderator_i_ + γ_5_InSys_it_ × Moderator_i_ + γ_6_TreatD_i_ × InSys_it_ × Moderator_i_ + ΩX_i_ + ε_it (2)

In equation 2, the variables have the same meaning as in equation 1, and *Moderator*_i_ refers to the moderators in this study including organizational rules and workload per physicians, the risk category of illness, and the clinical title of physicians.

## Results

### The Impacts of Prescription Automatic Screening System on Errors of Prescription and Medical Treatment Costs

This paper estimated a series of alternative models to measure the results and statistical fit of our DID model. We used a basic DID model without any control variables in model 1 while inserting control variables and physicians’ information into model 2. Next, we included moderators in model 3. According to the results presented in Multimedia Appendix 2, the result of fit statistic (*R*^2^) was seen to increase from model 1 to model 3 toward every variable, which supports the validity of the results.

With reference to the error of prescription, the results in Multimedia Appendix 2 indicate that the parameter of interactions between system onset and time lapse was continuously significant and negative from model 1 to model 2 (beta=–.246 *P*=.001; beta=–.257 *P*<.001). This indicates that physicians withdraw fewer times of prescriptions in hospital after system use. With the control variables to measure validity, the interaction variable parameter was still significantly negative in model 2 (95% CI –0.40 to –0.11; *P*<.001), indicating a negative impact of the system on the error of prescription. With reference to medical treatment costs, the results in Multimedia Appendix 2 show that the parameters of interaction between the system and cost are continuously significant and negative from model 1 to model 2 (beta=–.371 *P*=.007; beta=–.389; *P*=.007). With the control variables to measure the validity, the interaction variable parameter was still significantly negative in model 2 (95% CI –0.75 to –0.12; *P*=.007). This illustrates that PASS used in group A helped in lowering the costs for patients’ hospital stay, thus reflecting a lowered medical burden. However, according to *R*^2^, the model in this study could explain more about physicians’ medical performance.

### The Impact of Individual and Organizational Pressure

The effects of PASS implementation for the different moderating variables and the results of the difference-in-difference- in-difference (DDD) model are presented in model 3 in Multimedia Appendix 2. Moderating results relatively impact the moderating variable on dependent variables based on the 2-way interactions of the treatment effects in the DDD model. Moreover, the effects of system implementation on the error of prescriptions will differ depending on whether physicians belong to the department having PASS-related organizational rules. The parameters of interactions between the implementation of organizational rules and treatment effect (Treatment×InSys×Ins_pres) in model 3 (Multimedia Appendix 2) showed that PASS has fewer effects on prescription errors when physicians perceive high pressure from organizations (95% CI 0.07-0.55; *P*=.01). With respect to the medical treatment costs, the parameter of the interaction between workload and treatment effect (Treatment×InSys×Workload) was positively significant (95% CI 0.18-0.39; *P*<.001).

### Additional Analysis

**Figure 6:**
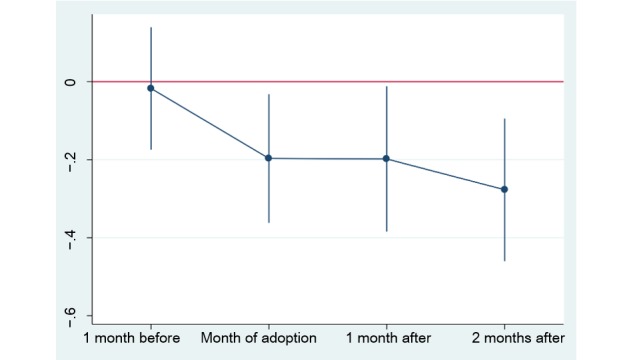
Time trends relative to month of adoption of the Prescription Automatic Screening System (PASS).

To examine the differences in hospitals before the implementation of PASS, we constructed the parallel trend test. Specifically, we added 2 indicator variables for each month before the system change, 3 indicators for each month after the system change, and the interaction terms of indicators and treatment. We chose 2 months before PASS as the baseline; the final results are presented in Table 2. According to the results in Table 2, there are no significant differences between the 2 hospitals before PASS implementation. Significant decrease in cost was observed in the month of adoption. The results also showed that the overall costs continued to decrease after the system change. We visualized this pattern in Figure 6.

**Table 2:** Results of parallel trend test.

Variables	Ln (cost)
Treatment	–0.286^a^
1 month before	–0.016
Month of adoption	–0.196^a^
1 month after	–0.197^b^
2 months after	–0.276^a^
Title_dummy1	0.129
Title_dummy2	0.067
Title_dummy3	0.191^b^
Ln(Workload)	–0.071
Gender	0.257^a^
_cons	9.244^c^
*R* ^2^	0.097

^a^*P*<.05.

^b^*P*<.01.

^c^*P*<.001.

## Discussion

### Prescription Automatic Screening System Reduces Prescription Errors and Medical Treatment Costs

Because the interaction of *Treatment* and *InSys* is negatively significant, applying PASS will reduce the prescription errors (beta=–.246, *P*=.001; beta=–.257, *P*<.001) and medical treatment costs (beta=–.371, *P*=.007; beta=–.389, *P*=.007). The results are consistent with the previous study that HISs decrease the prescription errors [14] and medical treatment costs [29]. As the primary role of IT, as applied to hospitals, the PASS system integrates knowledge derived from diverse individual specialists to support prescription-making decisions. This shortens the time in the treatment process and improves the effectiveness of using IT to support the diagnoses. Moreover, these findings highlight the various roles of IT to promote more appropriate coordination among individuals within the hospital setting, which could be used to improve the quality of health care. However, IT does exert a variety of effects when applied to different environmental considerations.

### Individual Pressure Presents No Impact

Contrary to the hypothesis, the moderators, risk categories (beta=.057, *P*=.33) and clinical title (beta=.272, *P*=.29; beta=.190, *P*=.47; beta=.200, *P*=.47) exerted insignificant effects, which is inconsistent with previous studies [30]. This may be due to the features of the medical domain; physicians will focus high attention on patients’ health conditions regardless of whether patients stay in high- or low-risk conditions. Further referring to the moderating effect of physicians’ title, the effects of the system on mitigating the anchoring bias are quite low because this factor will also influence the physicians’ behavior for physicians with both high and low clinical statuses. Hence, in the context of health care, personal characteristics will not limit the effects of the intelligent diagnosis system, which manifests a significant potential if hospitals could deploy more IT into their protocols to provide supplemental support for physicians’ decisions.

### Workload and Implementation of Organizational Rules Related to Usage of Prescription Automatic Screening System Decrease the Impact of Prescription Automatic Screening System

The parameters of interactions between organizational rules and the main model show that PASS has fewer effects on prescription errors when physicians perceive high pressure from organizations (95% CI 0.18-0.39; *P*<.001). With respect to the medical treatment costs, the parameter of the interaction between workload and main model is positively significant (95% CI 0.07-0.55; *P*=.01). The findings of the moderating effect indicate that environmental factors affect how IT alters users’ performance. Previous literature depicted an insignificant impact concerning related IT systems on physicians’ performance [24]. However, the findings of this study expanded on the previous model and proved that organizational rules might help clarify that the impact of IT will decrease when physicians perceive high pressure from organizations [31]. These results highlight the critical roles of management within organizations when they adopt IT in the workplace. The findings also emphasize that further exploration is needed to determine why pressure tends to eliminate the impact of IT from a psychological perspective.

Concerning the medical treatment costs, the results of the moderating effects related to the workload indicate that when physicians have more patients awaiting treatment, the effects of IT will decrease. This may be due to the fact that a high level of work pressure motivates physicians to depend more on their knowledge and experience, which will then lead them to ignore or disregard important alerting information, which is consistent with the previous study [32]. On the basis of this premise, the performance will not lead to significant differences even after the hospital deploys the PASS system.

In general, when hospitals adopted the IT system to enhance medical performance, they also implemented a corresponding policy designed to increase the effectiveness of IT usage. However, based on our findings, when the policy places too much pressure on the physicians, it will have a paradoxical result. Hence, our findings provide some significant new insights for policy implementation in hospitals, such as how to appropriately balance the policy between IT and organizational management protocols and how to effectively enact the evaluation criteria with regard to physicians’ performance.

### Strengths of This Study

In this study, we examined the impact of the PASS system by conducting a quasi-experiment, which could help eliminate the effects from various confounding factors and further highlight the causality between PASS and medical performance. According to the previous study, the impact of PASS may be quite varied based on the level of pressure exerted by the organizational environment. Through several cooperative interviews with physicians and administrators in hospitals, we obtained detailed information relating to the physicians’ attitudes and actual usage of PASS as further guidance in the exploration of environmental impact. Thus, we were able to evaluate how PASS plays its role in hospitals. We firmly believe these findings will provide practical suggestions for hospitals and their administration to garner a higher level of performance from their workforce after deploying the related systems.

### Limitations and Directions for Future Research

This study has 2 limitations. First, because some of the moderating effects in this study are insignificant, further exploration of these factors (ie, title and risk) is necessary, particularly, when emerging technologies (eg, artificial intelligence) are considered for use in hospitals. The problems to mitigate the new uncertainty, which derives from the new adoption behavior, are critical to improving the effectiveness of implementing HIS. To ascertain extended performance from PASS, attention must be paid to more categories of different hospitals with different characteristics. Second, based on the preliminary investigations, we need to proceed to provide a more specific classification of alerts, that is, physicians’ decision stages. This process requires further discussions with physicians, and the results will provide support for hypotheses on physicians’ performance and decision stages. We are aware that much more cooperation and data are required. In addition, although this paper examines the effects of the adoption of the PASS, the effective use of such a system will attract greater attention because of a higher quality of the treatment process, which we will investigate in our future research.

### Conclusions

This study found that PASS, a potential tool to integrate knowledge from various expertise, has positive effects on medical performance; however, organizational pressure raises a concern on the effectiveness of PASS. Specifically, we found that compared with individual pressure (eg, clinical title and disease risk), it is the pressure from the organization (eg, organizational rules and workload) that reduces the effectiveness of PASS. Hence, the strategies adopted by hospitals, which are used to improve the effectiveness of HIS implementation, may not work. The findings indicate that management in hospitals needs to balance the relationship between HIS implementation and policy making to augment the positive effects of HIS.

## Appendix

Multimedia Appendix 1 Details of interview results from 2 hospitals.

Multimedia Appendix 2 Results of regression analyses.

## Abbreviations

ADE: adverse drug events
ADR: adverse drug reactions
DDD: difference-in-difference-in-difference
DID: difference-in-differences
EMR: electronic medical record
GDP: gross domestic product
HIS: hospital information system
HIT: hospital information technology
IT: information technology
PASS: Prescription Automatic Screening System
PSM: propensity score matching
