# Supplementary material for: Exploring the Impact of the Prescription Automatic Screening System in Health Care Services: Quasi-Experiment
Source: JMIR Med Inform. 2019 Jun 14;7(2):e11663. doi: 10.2196/11663 (PMC6598418; doi:10.2196/11663)
Supplement: Multimedia Appendix 1 [file medinform_v7i2e11663_app1.docx]

## **Multimedia Appendix**

### Interviews

### *Data collection of interviews*

We conducted interviews with physicians, employees from information system department, and administrators of the two hospitals for a two-year period. During this time, we experienced many interactions with these people to gain a better understanding of the information on the implementation of the information systems in both hospitals, in relation to auxiliary systems.

To be specific, the data-gathering techniques included interviews with three physicians and two nurses from different medical departments, four employees from the information system department, three administrators from two hospitals. The questions in the interview with physicians involved in the IT use of HIS, primarily related to both EMR and PASS, e.g., How do you use the EMR in your daily work? Do you notice the alerting information from PASS when you are writing a prescription? What are the possible reasons behind your usage behaviors? Administrators were also interviewed to ascertain their roles in HIS use. The interview questions included the following: “*How is the PASS being used by the physicians in your department? How about the outcomes after deploying the PASS?*”

The interviews were open-ended and interactive by which could encourage respondents to provide more details or examples related to the specific cases, in particular for the points relevant to the topic of this research. Then, the viewpoints from about 30 minute interview were documented to themes pertinent to the initial dataset of this study. By coding and analyze the text, the results updated the theoretical framework.

Table A1 Respondents of interviews

| Position | Number | Department | Number |
| --- | --- | --- | --- |
| Chief physician | 1 | Obstetrics-gynecology | 3 |
| Associated chief physician | 0 | Endocrinology department | 3 |
| Physician | 2 | Neurology department | 2 |
| Nurse | 2 |  |  |
| Administrator | 3 |  |  |
| IT employee | 4 |  |  |

### *Results of interviews*

Table A2 Results of interviews detail

| Findings | Conclusion |
| --- | --- |
| Attitude toward EMR and PASS |  |
| In the light of the dialogue with physicians and nurses, the roles of EMR and PASS during their work have a great deal of controversy and divergent. For one hand, physicians posited that EMR could help track the history information of patients’ medical record, and PASS offers the details of medicines including dosage, instruction and elemental compositions which support them to make a decision.  On the other hand, physicians also claimed that EMR and PASS cause many limitations which retard their work efficiency. Through further communication with physicians and nurses, the dark side of EMR and PASS mainly manifests in the emergency work when the workload is high. | - PASS and EMR are useful for both physicians and nurses. - The workload will stimulate the dark side of PASS. - PASS will assist physicians’ decision-making. |
| For example, one of the physicians mentioned that: *“…I believe the EMR facilitates lots of conveniences to my routine work, however, when there are lots of patients waiting for the medical services or there is an emergency case, the reminding information from PASS will impede my work efficiency.”* Additionally, nurses generally show the satisfaction with EMR. Hence, even though EMR improving the work efficiency, decision supports from IT are more critical for physicians while IT impacts vary with the various workload. |  |
| Actual use toward EMR and PASS |  |
| The interviewed physicians revealed that they would heed the reminders and follow the instructions from PASS while most of the instructions are reasonable, primarily when the prescription activates the alerts for antibiotics. Similar to answers from employees of information system department, PASS will recommend the optimal solutions to physicians to assist a decision. However, one point physicians mentioned is that the alerting information may be inconsistent with the prior experience of physicians. Since employees from information system departments argue that the database of medicine information is constructed depending on the national standardize database, the standard information generally does not adapt to every physician, in particular for the experienced one. Hence, this conflict phenomenon implies that the impacts of PASS on physicians’ behavior will differ among individuals with different experiences. | - Physicians will follow the alerting information when they are making a decision. - The alerting information may not be appropriate for the special cases. - Physicians have more experiences have higher possibility to have different opinions with the alerting information. |
| Managerial governance toward EMR and PASS usage |  |
| In addition to the in-process control of PASS, supervised physicians could gain access to post-process control by the total statistical usage of medicines, especially for antibiotics, which are governed by the strict rules. Hence, in general, the PASS alleviates health uncertainty and risk by means of strict monitoring from both organizational and technical. With respect to the governance of the organization, administrators indicate that statistical of the medicines in prescriptions is required every month with strict limitation. Hence, under the different strategy of management, the impacts of IT on performance will vary. | - From the view of administrators, physicians followed the instruction of alerting information to make a decision. - Hospitals will implement a related policy to guarantee the effectiveness of the system after deployed. |

### Robustness Checks

In our primary analysis, we used PSM-DID as our crucial identification strategy in Section 4 and used identical physicians working in hospital B. One possible concern with this approach is that the two hospitals may have different differences in strategy, essential equipment, and other aspects which will influence physicians’ behavior differently. We conduct the DID regression to determine if the difference in physicians’ behavior arises from the system or the external environment. On the basis of the results, treatment group presents the significant difference in pre- and post- period than the control group. However, the difference is more significant for the errors of prescription. The results prove the significant impacts of PASS on the physicians’ behavior. Further, we moved the launch time of PASS forward to the one month and three month before May, 2012 to examine the impacts of PASS. This method could also ensure that the results were not the result of unobservable factors present during certain periods and were robust to the specifications of the time windows, the results of which are reported in Table A3. We found that the DID estimates are insignificant in each of the above cases indicating the impact is from our study’s PASS and not other systems.

Table A3 Results of Robustness Checks

| Time period |  |  | |
| --- | --- | --- | --- |
|  | 2011/8-2012/1  2012/2-2012/7 | 2011/11-2012/4  2012/5-2012/10 | |
| Variables |  |  |  |
|  | Ln(Cost) | Ln(Error) | Ln(Cost) |
| *Treat* | -1.439^c^ | -0.125 | -0.224^a^ |
| *Insys* | 0.038 | 0.089 | -0.005 |
| *Treat*×*Insys* | 0.020 | -0.044 | 0.045 |
| ^a^*P*<0.05  ^b^*P*<0.01  ^c^*P*<0.001 | | | |

Since this study used the inpatients as the sample frame, the admission period will also influence the physicians’ behavior and the fee. Thus, this study further controls the length of stay of patients to eliminate the impacts from illness period. The results are reported in Table A4. When the model controls the length of stay, the results are still consistent with the previous findings which prove the robustness of the findings. The illness period will not influence the errors of prescription and fee of the prescription.

Table A4 Results of regression

| Variables |  |  |
| --- | --- | --- |
|  | Ln(Error) | Ln(Cost) |
| *Treatment* | -0.463^c^ | -0.329^c^ |
| *InSys* | 0.021 | 0.221^b^ |
| *Treatment*InSys* | -0.255^c^ | -0.282^a^ |
| *Gender* | -0.076^a^ | -0.023 |
| *Ins_type_dummy1* | -0.236 | 0.132 |
| *Ins_type_dummy2* | -0.025 | 0.287 |
| *Ins_type_dummy3* | -0.178 | 0.025 |
| *Ln(length of stay)* | 0.049 | 0.864^c^ |
| _cons | 1.940 | 6.795 |
| R^2^ | 0.591 | 0.512 |
| ^a^*P*<0.05,  ^b^*P*<0.01,  ^c^*P*<0.001 | | |
