# Supplementary material for: Exploring the Impact of the Prescription Automatic Screening System in Health Care Services: Quasi-Experiment
Source: JMIR Med Inform. 2019 Jun 14;7(2):e11663. doi: 10.2196/11663 (PMC6598418; doi:10.2196/11663)
Supplement: Multimedia Appendix 2 [file medinform_v7i2e11663_app2.docx]

Table 1. Results of regression.

| Variables | Models | | | | | |
| --- | --- | --- | --- | --- | --- | --- |
|  | Model 1  No control variables | | Model 2  Control variables | | Model 3 DDD | |
|  |  | |  | |  | |
|  | Ln(Cost) | Ln(Error) | Ln(Cost) | Ln(Error) | Ln(Cost) | Ln(Error) |
| Treatment | –0.244^b^ | –0.466^c^ | –0.331^c^ | –0.455^c^ | –0.778^b^ | –0.457^b^ |
| InSys | 0.311^b^ | 0.041 | 0.253^b^ | 0.032 | 0.248 | 0.459^b^ |
| Treatment*InSys | –0.371^b^ | –0.246^b^ | –0.389^b^ | –0.257^c^ | –1.012^a^ | –0.665^b^ |
| Ln(Workload) | — | — | — | — | –0.296^b^ | 0.308^b^ |
| Title_dummy1 | — | — | — | — | –0.008 | –0.096 |
| Title_dummy2 | — | — | — | — | –0.090 | 0.004 |
| Title_dummy3 | — | — | — | — | –0.349^c^ | –0.050 |
| Ln(Ins_pres) | — | — | — | — | 0.074 | –0.022 |
| Risk | — | — | — | — | 0.145^b^ | –0.027 |
| Treatment×  Workload | — | — | — | — | 0.111 | –0.060 |
| InSys×Workload | — | — | — | — | 0.025 | –0.141^c^ |
| Treatment×Risk | — | — | — | — | 0.037 | 0.084^a^ |
| InSys×Risk | — | — | — | — | –0.026 | 0.043 |
| Treatment×Ins_pres | — | — | — | — | –0.087 | 0.063 |
| InSys×Ins_pres | — | — | — | — | –0.317^a^ | 0.063 |
| Treatment×Title_dummy1 | — | — | — | — | 0.312 | 0.093 |
| Treatment×Title_dummy2 | — | — | — | — | 0.146 | –0.008 |
| Treatment×Title_dummy3 | — | — | — | — | 0.334^a^ | 0.158 |
| InSys×Title_dummy1 | — | — | — | — | –0.030 | 0.077 |
| InSys×Title_dummy2 | — | — | — | — | –0.003 | –0.023 |
| InSys×Title_dummy3 | — | — | — | — | –0.014 | 0.059 |
| Treatment×InSys×Risk | — | — | — | — | –0.007 | –0.088 |
| Treatment×InSys×Ins_pres | — | — | — | — | 0.236 | 0.336^c^ |
| Treatment×InSys×Workload | — | — | — | — | 0.441^b^ | –0.140 |
| Treatment×InSys  ×Title_dummy1 | — | — | — | — | 0.087 | — |
| Treatment×InSys  ×Title_dummy2 | — | — | — | — | 0.090 | 0.017 |
| Treatment×InSys  ×Title_dummy3 | — | — | — | — | 0.144 | 0.039 |
| Gender | — | — | 0.391^c^ | 0.005 | 0.386^c^ | –0.064^a^ |
| Constant | 9.052 | 1.837 | 8.822 | 1.694 | 9.345^c^ | 1.429^c^ |
| R^2^ | 0.130 | 0.510 | 0.270 | 0.530 | 0.388 | 0.679 |

^a^*P*<.05.

^b^P<.01.

^c^*P*<.001.
